# ADAM and ADAMTS Proteins, New Players in the Regulation of Hepatocellular Carcinoma Microenvironment

**DOI:** 10.3390/cancers13071563

**Published:** 2021-03-29

**Authors:** Nathalie Théret, Fidaa Bouezzeddine, Fida Azar, Mona Diab-Assaf, Vincent Legagneux

**Affiliations:** 1 Univ Rennes, Inserm, EHESP, Irset (Institut de Recherche en santé, Environnement et Travail)-UMR_S1085, University of Rennes 1, 35000 Rennes, France; Fida.azar@inrae.fr (F.A.); vincent.legagneux@univ-rennes1.fr (V.L.); 2 Molecular Cancer and Pharmaceutical Biology Laboratory, Faculty of Sciences II, Lebanese University Fanar, 1500 Beirut, Lebanon; Fidaa.bouezzeddine@ul.edu.lb (F.B.); mdiabassaf@ul.edu.lb (M.D.-A.)

**Keywords:** hepatocellular carcinoma, fibrosis, metalloproteinase, disintegrin

## Abstract

**Simple Summary:**

Members of the adamalysin family are multi-domain proteins involved in many cancer-related functions. In this review, we will examine the literature on the involvement of adamalysins in hepatocellular carcinoma progression and their importance in the tumor microenvironment where they regulate the inflammatory response and the epithelial–mesenchymal transition. We complete this review with an analysis of adamalysin expression in a large cohort of patients with hepatocellular carcinoma from The Cancer Genome Atlas (TCGA) database. These original results give a new insight into the involvement of all adamalysins in the primary liver cancer.

**Abstract:**

The tumor microenvironment plays a major role in tumor growth, invasion and resistance to chemotherapy, however understanding how all actors from microenvironment interact together remains a complex issue. The tumor microenvironment is classically represented as three closely connected components including the stromal cells such as immune cells, fibroblasts, adipocytes and endothelial cells, the extracellular matrix (ECM) and the cytokine/growth factors. Within this space, proteins of the adamalysin family (ADAM for a disintegrin and metalloproteinase; ADAMTS for ADAM with thrombospondin motifs; ADAMTSL for ADAMTS-like) play critical roles by modulating cell–cell and cell–ECM communication. During last decade, the implication of adamalysins in the development of hepatocellular carcinoma (HCC) has been supported by numerous studies however the functional characterization of most of them remain unsettled. In the present review we propose both an overview of the literature and a meta-analysis of adamalysins expression in HCC using data generated by The Cancer Genome Atlas (TCGA) Research Network.

## 1. Adamalysins Are Multidomain Proteins with Multiple Functions

**Figure 1:**
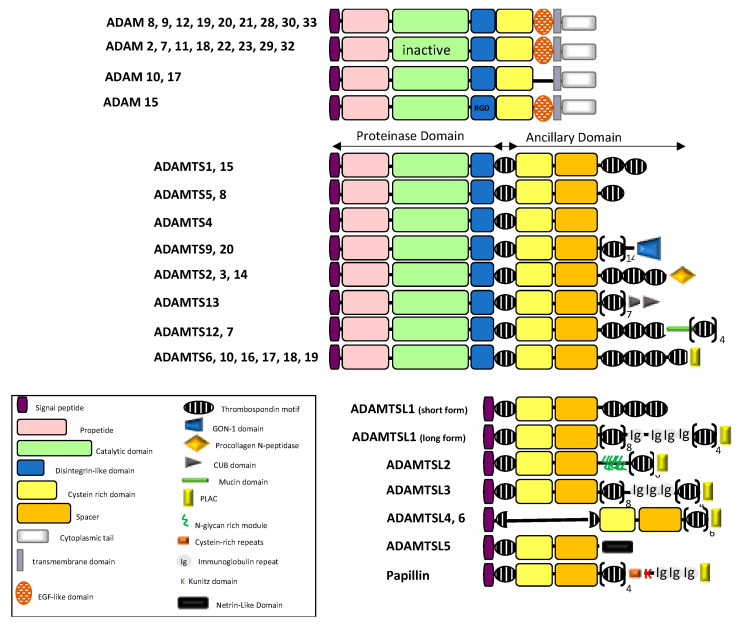
Schematic diagram illustrating the domain organization of the ADAM/TS-TSL family members (adapted from [2,3,5]).

Adamalysins belong to the zinc protease superfamily and are characterized by a multidomain organization that confers multiple functions [1]. Numerous reviews have already detailed the classification and functions of ADAM and ADAMTS proteins (ADAM for a disintegrin and metalloproteinase; ADAMTS for ADAM with thrombospondin motifs; ADAMTSL for ADAMTS-like) and their implication in physiological and pathological processes [2,3]. The ADAM–TS–TSL family consists of 20 ADAMs, 19 ADAMTS and 7 ADAMTSLs (1 to 6 and papillin) in human. These proteins share a multi-domain organization including a signal peptide, a pro-peptide and metalloprotease, disintegrin and cysteine-rich domains (Figure 1). While ADAMs are membrane proteins characterized by additional epidermal growth factor (EGF)-like, transmembrane and cytoplasmic domains, ADAMTS members are secreted proteins characterized by an ancillary domain containing a thrombospondin type 1 repeat (TSR), a spacer domain and except for ADAMTS4, additional motifs including TSRs. Unlike ADAM and ADAMTS, ADAMTSL genes encode secreted proteins that lack catalytic and disintegrin domains and are likely involved in ECM assembly.

**Figure 2:**
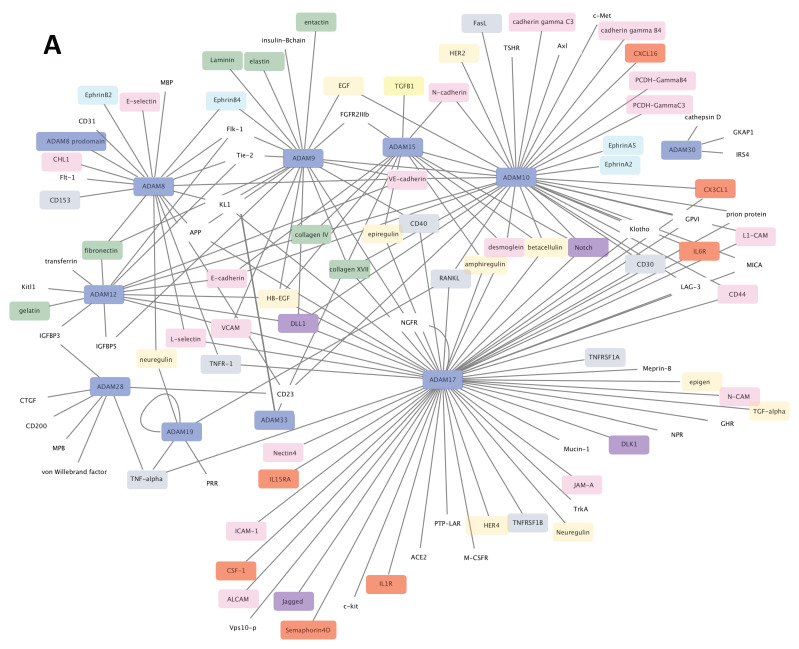

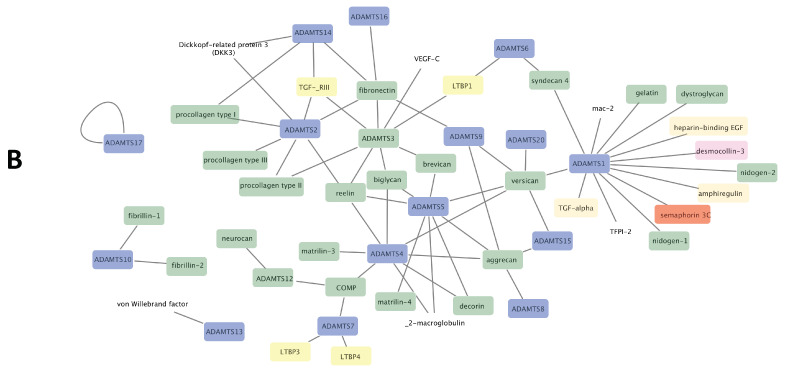
Substrates networks for ADAM (**A**) and ADAMTS (**B**). Generated from data reported in [5,17] and plotted using cytoscape software platform (https://cytoscape.org/, accessed on 14 January 2021). Colors of nodes: blue, ADAM/ADAMTS; green, extracellular matrix components; red, cytokine/chemokine; pink, adhesion/junction molecules; grey, TNF superfamily components; purple, Notch signaling components; yellow, TGF-β signaling components; turquoise, ephrin signaling components. The remaining uncolored nodes correspond to miscellaneous functions.

A major feature of ADAM and ADAMTS proteins resides in their catalytic properties. ADAM metalloproteinases were rapidly named sheddases since most of their substrates were membrane-bound precursors. However only 12 ADAMs including ADAM8, 9, 10, 12, 15, 17, 19, 20, 21, 28, 30 and 33 have a functional catalytic site and two of them (ADAM20 and 21) have no known substrates (reviewed in [4,5]). Most of substrates for the mentioned 12 ADAMs are growth factors, chemokines, adhesion molecules and their receptors and only very few extracellular matrix components were reported (Figure 2A). While there is no specific substrate repertoire for each ADAMs, the phylogenic neighbors ADAM10 and 17 share many substrates including Notch and its ligand Delta-like 1 (DLL1), TNF receptor (CD30), the receptor activator of nuclear factor κ-B ligand (RANKL), the receptor to IL-6 and adhesion molecules such as CD44, desmoglein and L1CAM. One important feature that characterize ADAM9, 10, 12, 15 and 17 is their ability to interfere with EGFR- and Notch-dependent signaling pathways through processing of either the receptor or the ligands [6,7,8]. The regulation of these signaling pathways can occur in an ADAM’s catalytic-independent way such as the ADAM12-dependent activation of TGF-β signaling pathway [9] and the disintegrin domain-dependent binding for integrin-mediated signaling [10].

Splice variants have been reported for ADAM9 and ADAM12 giving rise to secreted forms with implication in cancer cell invasion. Similarly, ADAM11, ADAM28 and ADAM33 have short spliced variants lacking transmembrane and cytoplasmic tails while variants for ADAM15 and ADAM22 affect only cytoplasmic domain. ADAM19 and ADAM33 variants are characterized by alternative splicing within the pro-, metalloprotease and disintegrin domains.

ADAMTS proteins are proteases which have been historically classified according to their substrates: the aggrecanase and proteoglycanase (ADAMTS1, 4, 5, 8, 9, 15 and 20); the pro-collagen N-propeptidases (ADAMTS-2, 3 and 14); the COMP proteinases (ADAMTS7 and 12) and the von Willebrand factor proteinase (ADAMTS13). For a long time, the remaining ADAMTS were considered as orphan enzymes [2,3,11] however, identification of several new substrates complicated the picture. A large screening for ADAMTS2, 3, and 14 substrates identified components of TGF-β network (the latent TGF-β binding proteins LTBP1 and 2, the Transforming Growth Factor Beta Receptor 3, TGFBR3 and Decorin that binds active TGF-β) as well as extracellular matrix proteins such as fibronectin [12]. Fibronectin was also recently identified as a substrate of ADAMTS16 [13] and ADAMTS9 [14] thereby supporting evidence for the implication of ADAMTS9 and ADAMTS16 in ECM assembly and turnover. Using Terminal Amine Isotopic Labeling of Substrates (TAILS) method, Colige et al. recently identified new substrates repertoire for ADAMTS7 including LTBPs, fibronectin, and fibrillin-2 [15], the latter being also identified as a new substrate for ADAMTS10 [16]. The absence of ADAM-specific motif in the substrates led to the complex identification of ADAM substrates. Based on the recent review from [17], we built the substrates network of ADAMTS proteins giving rise to a new classification based on functional proximity (Figure 2B). An important feature of ADAMTS proteins is the non-catalytic ancillary domains which support substrate recognition [18] and share homologies with ADAMTS-like proteins. The critical contribution of ADAMTSs and ADAMTSLs in microfibril formation, stabilization and functions is now well established [19]. In accordance with this, mutations in ADAMTS2, 3, 10, 13, 17, 20 and in ADAMTSL2 and ADAMTSL4 are associated with Mendelian disorders affecting ECM assembly and regulation of growth factors bioavailability [20,21,22].

Because of their multiple functions in regulation of cell–cell and cell–matrix communication, ADAM and ADAMTS proteins are key players in maintaining tissue homeostasis and their deregulation is associated with numerous biological processes including tissue remodeling, inflammation and cell migration. In that context, the role of ADAMs [5,23] and ADAMTSs [24,25] in cancer have been widely documented especially in angiogenesis [26], their contribution to the tumor environment [27] and their use as targets for the treatment of cancer [28]. Here we will focus on hepatocellular carcinoma, the most common primary liver cancers that mostly develop in the context of chronic liver disease.

## 2. Adamalysins in Hepatocellular Carcinoma

### 2.1. Adamalysin Expression and Association with Overall Survival

In 2010, Mazzocca et al. wondered about the «real pathogenic link» between ADAM proteins and tumorigenesis and progression of hepatocellular carcinoma [29]. There is no longer any doubt today that adamalysins are involved in the development of HCC. Increased expression of many Adamalysins including ADAM8 [30,31], ADAM9 [32,33], ADAM10 [34,35], ADAM12 [36] and ADAM17 [37] was reported in HCC and associated with tumor progression. Additional studies demonstrated that over-expression of these ADAMs promoted proliferation of HCC cells and was predictive of poor survival outcomes. In parallel, miRNA-dependent down-regulation of ADAM9 [38,39,40], ADAM10 [41,42,43,44,45,46] and ADAM17 [47,48,49,50] was associated with reduced tumor progression, inhibition of cell proliferation and invasion and chemotherapy sensitization of HCC cells. The liver-specific miR-122 that targets both ADAM10 and ADAM17 is decreased in patient with HCC and was recently proposed as a biomarker for hepatocellular carcinoma related to hepatitis C virus infection [51]. By contrast, the long non coding LIN0551 competes miR122 in regulating ADAM10 thereby promoting HCC cell proliferation and invasion [52]. Alternatively, spliced variants of ADAM12 and ADAM9 were identified in activated hepatic stellate cells and among those of ADAM9, the short form (ADAM9-S) promoted cancer cell invasion [36,53]. In addition to these spliced variants, single nucleotide polymorphism (SNP) variants of ADAM10 were sequenced from patients with HCC and some of them including rs514049 and rs653765 variants were associated with a higher risk of developing metastases [54]. Besides the well documented ADAM9, ADAM10 and ADAM17 genes, the over-expression of ADAM18, ADAM21 and ADAM32 was more recently associated with HCC invasion [55,56], however the molecular mechanisms that were involved remain unexplored.

To have a complete view of the expression of all adamalysins in HCC, we took advantage of RNAseq data generated by The Cancer Genome Atlas Program (TCGA) Research Network: https://www.cancer.gov/tcga, accessed on 14 January 2019. Normalized expression values were extracted via the ‘International Cancer Genome Consortium” website (https://icgc.org/, accessed on 11 July 2016). The Cancer Genome Atlas Liver Hepatocellular Carcinoma (TCGA-LIHC) data collection contains 364 LIHC samples including 48 paired hepatocellular carcinoma and adjacent non-tumor tissues. Of course, RNA sequencing data provide new valuable information although do not permit to evaluate adamalysin activity. We also analyzed the prognosis value of adamalysins expression in TCGA-LIHC samples using Kaplan–Meier plotter online tool (http://kmplot.com/analysis/, accessed on 1 September 2020). Comparison of adamalysin expression levels between tumor (T) and adjacent non-tumor (NT) tissues and survival analyses are given in Table 1, left and right panels, respectively. Note that the same TCGA-LIHC samples were recently used to propose ADAM9 as a biomarker in advanced HCC [57]. In agreement with previous reports, we observed that expression levels of ADAM9, ADAM10, ADAM12 and ADAM17 were increased in tumor versus adjacent non tumor tissues except for ADAM10 that was non-significant, all were associated with poor prognosis. Importantly, we reported for the first time an up-regulation of ADAM15, ADAM21, ADAM22, ADAM23 in tumors compared with adjacent non tumor tissues, however only ADAM15 is indicative of worse prognosis (0.007). The very high significant increase expression of ADAM15 in tumor (8.2 × 10^−19^) is in agreement with the up-regulation of ADAM15 previously reported in other cancers including breast, lung, prostate and bladder cancers [23]. The role of ADAM15 in invasion and metastasis involves the regulation of claudin-1 and its interaction with the tight junction proteins ZO1 and ZO2 [58]. Interestingly, these tight junction proteins also play a critical role in hepatitis C virus infection [59]. In addition, ADAM15 is known for its role in inflammation [60] suggesting its contribution to HCC development.

The implication of ADAMTS proteins in cancer was also previously reported and both anti-tumor and pro-tumor functions were described [24,25,61]. In the liver, expression levels of ADAMTS1 were firstly reported as being lower in human HCC samples than in the underlying cirrhotic tissue [62,63]. Low expression of ADAMTS1 in HCC compared with healthy livers was next reported [62,64]. More recently, up-regulation of ADAMTS1 expression was observed in HCC cells upon stimulation by either the inflammatory cytokines IL-1β and IL-6 [65] or by hypoxia [66]. ADAMTS1 was also reported in non-parenchymal cells including endothelial cells [67] and activated hepatic stellate cells [68]. Using the NT/T paired sample data set from TCGA database, we confirmed decreased expression of ADAMTS1 in tumor samples compared with the non-tumor adjacent tissues (*p* = 0.000059) suggesting anti-tumor functions. By contrast, the high expression of ADAMTS1 in cirrhotic tissues [63], the up-regulation of ADAMTS1 in liver fibrosis [69] and the implication of ADAMTS1 in TGF-β activation in activated hepatic stellate cells (HCS) [68] suggest a role of ADAMTS1 in liver fibrosis that might favor tumor onset.

Low expression levels of ADAMTS5 [70] and ADAMTS8 [71] were associated with poor prognosis of patients with HCC. ADAMTS8 inhibited proliferation and favored apoptosis of HCC cells [71]. Using TCGA data, we confirmed that low expression of ADAMTS8 was associated with overall survival however, we observed the increased expression of ADAMTS5 in tumor and its association with worse survival (Table 1). We also observed a significant increase in ADAMTS9 expression in tumors compared with adjacent non tumor tissue and an association between high expression and worse prognosis. However, ADAMTS9 was previously suggested to act as a suppressor of tumor since increased expression of microRNA-32 [72] and long non-coding RNA ADAMTS9-AS1 [73] that both target ADAMTS9, were correlated with decreased patient survival, and increased proliferation and invasion, respectively. Our own analysis showed also increased expression of ADAMTS7, 14 and 18 in tumors compared with adjacent non tumor tissues and high expression in tumors was associated with worse prognosis (Table 1). In accordance with this observation, the pro-tumor role of ADAMTS7 was recently supported by its identification in a signature of cancer stem cells that contribute to the development and therapeutic resistance of HCC [74]. ADAMTS14 and 18 have not yet been documented in liver cancers. By contrast ADAMTS13 was widely studied since liver is the main source of ADAMTS13 in human and changes in ADAMTS13 activity was associated with liver disease [75]. Several reports showed a decrease of ADAMTS13 activity either in cirrhosis [76] or in plasma of patients with viral [77] or alcohol [78] hepatitis. However, Lisman et al. [79] did not report significant difference in ADAMTS13 expression levels between cirrhosis and control livers and Ikeda et al. [80] recently showed that high plasma ADAMTS13 activity was a risk factor for HCC development in patients with chronic hepatitis B and C. Ikeda et al. [80] suggested that these discrepancies might be related to the difference in hepatitis activity and wound healing in selected patients. More recently, Takaya et al. proposed to use the ratio between ADAMTS13 activity and its substrate the von Willebrand factor as a biomarker either for the response to sorafenib treatment [81] or early diagnosis of HCC [82]. In line with this controversy, Kume et al. [83] showed that apoptosis of HSCs, the major ADAMTS13-producing cells [84] contributes to decrease ADAMTS13 levels in dimethylnitrosamine-treated rats but not in (CCl4)-treated rats which are characterized by the absence of HSC apoptosis and up-regulation of ADAMTS13 [85]. Also, a novel spliced ADAMTS13 transcript was found in both hepatic stellate cells and HCC cell lines [86] suggesting that tumor cells could also participate in expression of ADAMTS13 in HCC. Using paired HCC samples from TCGA database, we observed a very significant decrease of ADAMTS13 expression in tumor compared to adjacent non tumor tissues (3.8 × 10^−21^), however ADAMTS13 expression was not predictive for prognosis in the whole unpaired cohort (Table 1).

The balance between pro-tumor and anti-tumor effects of ADAMTSs has been partly attributed to their dual role in angiogenesis, a critical process in cancer progression [26]. The anti- and pro-angiogenic effects of ADAMTS1 were widely documented elsewhere [87] acting either as protease or in a catalytic-independent way. In hepatocellular carcinoma, only ADAMTS5 was suggested to inhibit angiogenesis through down-regulation of VEGF in HCC cells [70]. Variants of ADAMTS14 [88] and ADAMTS5 [89] were associated with susceptibility to hepatocellular carcinoma in a Chinese Han population, however the functional effects of these polymorphisms that occur near the anti-angiogenic TSR domain were not demonstrated.

The implication of ADAMTSL proteins in cancer is still largely undocumented. Changes in expression levels of ADAMTSL3 and ADAMTSL5 were reported in hepatocellular carcinoma, however opposite roles were suggested. ADAMTSL3 was identified as a tumor suppressor gene since down-regulation of ADAMTSL3 is associated with poor overall survival and predicted poor relapse-free survival [90]. At the opposite end, high ADAMTSL5 expression was associated with hypermethylation in HCC and a shorter overall survival of patients [91]. Our own analysis of HCC samples from the TCGA database provides new information on ADAMTSL expression levels in these samples (Table 1). In agreement with previous studies, we observed decreased ADAMTSL3 expression in tumors compared with adjacent non tumor tissues and low ADAMTSL3 expression was associated with a worse prognosis. By contrast ADAMTSL5 expression was increased in tumor versus adjacent non tumor tissues. In addition to these expected observations, our analysis now revealed that low expression levels of ADAMTSL1, ADAMTSL2 and ADAMTSL4 were predictive of worse prognosis suggesting similar suppressor role for the four ADAMTSLs (1–4) in HCC. These observation in HCC are consistent with previous reports in other cancers. Decreased ADAMTSL3 expression was reported in colon carcinoma [92] and ADAMTSL5 was over-expressed in melanomas [93]. In addition, increased methylation in ADAMTSL5 was associated with chemo-resistance in Acute B Lymphoblastic Leukemia patients [94]. Very few information about ADAMTSL1, 2 and 4 is available in cancers, however two SNPs in ADAMTSL1 gene were associated with early-onset disease-free survival in breast cancers [95] and ADAMTSL1 was shown to regulate chondrosarcoma cell proliferation [96]. More recently ADAMTSL4 expression was identified in glioma stem-like cells contributing to a five gene signature associated with bad prognosis of patients with primary glioblastoma, suggesting that ADAMTSL4 might have contrasting roles depending on cell origin [97].

### 2.2. Implication of Adamalysins in Inflammation and HCC Progression

**Figure 3:**
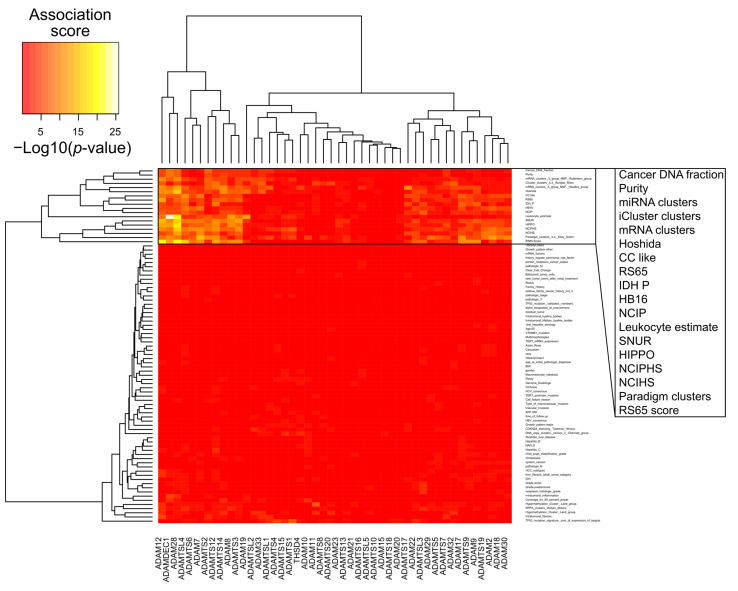
Association between expression levels of Adamalysin genes and molecular features of hepatocellular carcinoma (HCC) samples (TCGA-LIHC primary tumors, 294 samples) extracted from the Cancer Genome Atlas Research Network [98]. Heat map shows association scores expressed as −Log10(*p*-values). For quantitative features, *p*-values result from a Pearson’s correlation test between feature values in HCC samples and the corresponding expression values for a given gene. For qualitative values (HCC subtypes), *p*-values result from a non-parametric distribution test (Kruskal-Wallis) of the expression values for a given gene among HCC subtypes (clusters).

To further characterize biological processes associated with adamalysins expression in hepatocellular carcinoma, we used the molecular and clinical feature previously described for TCGA HCC samples in [98]. As shown in Figure 3, the main molecular signatures associated with adamalysin expression are related to inflammation (leukocyte estimate), recurrence (SNUR, RS65.Sscore) and poor differentiation state (NCIHS, NCIPHS, cholangiocarcinoma-like). Because of their shedding activity that targets molecular actors of the immune system, the regulatory role of ADAM proteins in inflammatory processes have been widely documented [99]. Such activities particularly make sense in HCC since this cancer mainly develops in the context of chronic liver inflammation leading to fibrosis which in turn promotes the initiation and progression of tumors [100].

#### 2.2.1. Regulation of Pro-Tumorigenic Cytokines by Adamalysins in HCC

Among the targets of ADAMs, inflammatory cytokines such as tumor necrosis factor-α (TNF-a) and their receptors such as interleukin-6 receptor play critical roles in liver inflammation and HCC development [101]. Shedding of membrane-bound TNFα releases a soluble TNFa that binds to TNFRs to induce different signaling pathways leading either to cell survival and pro-inflammatory gene expression or to apoptosis and cell death [102]. In addition, the proteolytic cleavage of TNFR by ADAMs generates a soluble receptor that regulates TNFα bioavailability. In the liver, ADAM17 was directly involved in regulation of TNFα system in inflammation associated with different hepatic processes such as regeneration [103] and severe Alcoholic Hepatitis [104]. A link between inflammatory signals and pro-tumorigenic mechanisms in liver cells is supported by the ability of the inflammatory cytokine TNFα to induce activation of EGFR signaling. Argast et al. [105] firstly suggested the implication of ADAM17 in the proliferative effect of TNFα through the release of TGFα that activates EGFR in mouse hepatocyte cell lines. Similar mechanism was reported in human HCC cells where TNFα treatment induced increase of the EGFR ligand amphiregulin (AR) and this effect was abrogated by the ADAM17 inhibitor GM6001 [106]. ADAM17 was also involved in the regulation of IL-6-dependent signaling pathway that links chronic inflammation to HCC progression. Besides the classical pathway induced by IL-6 binding on its receptor, the proteolytic release of the soluble IL-6 receptor (sIL-6R) by ADAM17 induces a trans-signaling pathway, a mechanism allowing to sensitize cells that do not express IL-6R [107,108]. Increase of IL-6 and sIL-6R has been reported in cirrhosis and HCC [109,110] and Bergmann et al. demonstrated that the IL-6 trans-signaling mechanism is essential for the development of HCC [111]. Notch signaling is another pathway tightly regulated by adamalysins either through shedding of Notch receptor or its ligand [7,112]. The role of Notch signaling in the development of liver cancers has been recently reviewed [113,114] however, the direct implication of adamalysins in this pathway remains poorly documented, except for ADAM17. ADAM17-dependent Notch activation was involved in the induction of IL10 producing CD4 + T cells mediated by hepatocytes from Con A-pretreated mice contributing to the regulation of immune response [115]. Notch signaling plays also a regulatory role in maintenance of cancer stem-like cells (CSC) phenotype and Notch proteolytic activation by ADAM17 was implicated in promotion of liver CSCs upon iNOS over-expression [116]. Proteolytic activity of ADAM10 and 17 was also involved in the shedding of the receptor tyrosine kinase Met in HCC cells and hepatic stellate cells [117]. The authors further demonstrated a correlation between the soluble form of Met (sMet) and liver damage and inflammation. While sMet has been proposed as biomarker of cancer aggressiveness and bad prognostic [118,119,120], it was shown to down-regulate HGF signaling by trapping this growth factor and by preventing c-Met receptor dimerization [121].

#### 2.2.2. Role of Adamalysins in Tumor Escape from Immune Surveillance

ADAM-dependent shedding of the major histocompatibility complex class I-related chain A (MICA) expressed by tumor cells prevents recognition by the NKG2D receptor expressed by natural killer (NK) cells thereby contributing to evading anti-tumor immunity [122]. According with this, the multi kinase inhibitors sorafenib and regorafenib enhance natural killer (NK) cells cytotoxicity against HCC cells by reducing ADAM9 and ADAM10 expression [123,124,125]. The contribution of ADAM17 in MICA shedding in HCC cells is more controversial. Although ADAM17 was firstly unrelated to MICA shedding [124], more recent data showed that ADAM17 contributed to MICA shedding in HEPG2 and HEP3B cells [123,126]. At the opposite, ADAM28 was recently proposed to play a protective role against the dissemination of cancer cells by promoting the T cell immune response [127]. However, proliferation of liver cancer cells was inhibited by miR-574-3p through ADAM28 targeting thereby suggesting that ADAM28 promotes liver cancer [128]. The ambivalent functions of ADAM28 in cancer were recently reviewed in [129] where expression of ADAM28 in cancer cells contribute to their proliferation and migration while stromal expression of ADAM28 contributes to protective effects.

#### 2.2.3. Adamalysins Regulate Invasion in HCC

Among shedding activities associated with cancer, the importance of ADAM-dependent shedding of EGF-like ligands in proliferation and invasion of cells is widely documented [130,131]. In hepatocellular carcinoma, Itabashi et al. [132] firstly reported the suppression of angiotensin II-induced cell proliferation by targeting the AngII–EGFR cross-talk signaling mediated by ADAM9 and ADAM17. ADAM17 was further involved in the hypoxia-induced drug resistance of HCC cells through activation of EGFR/PI3K/Akt pathway [133]. The contribution of ADAM9 and ADAM17 to HCC proliferation and invasiveness was also supported by the suppressive effects of miRNAs that specifically target them [39,48,49,50]. More specifically, several adamalysins were associated with the epithelio–mesenchymal transition (EMT), a mechanism that favors proliferation, stemness, invasiveness, immune escape and resistance to anti-cancer treatments [134,135]. Different molecular mechanisms have been reported in ADAM-mediated EMT in liver. ADAM9 was involved in the IL-6 dependent EMT of HCC cell lines through interaction with the NADPH oxydase, thereby favoring ROS production [136]. This effect is linked to an increased expression of Snail, a major transcriptional driver of EMT, while the expression of other EMT-promoting factors such as Slug, Twist or Zeb were not affected suggesting that ADAM9 promotes a partial EMT. The contribution of ADAM17 in promoting EMT of HCC cells involves the activation of Notch signaling pathway, by increasing the proteolytic cleavage and release of the active Notch intracellular domain (NICD) [49]. In this study, pro-EMT effects of ADAM17 were antagonized by a specific micro-RNA (miR-3163) whose anti-EMT effects were associated with a decreased expression of EMT-promoting transcription factors (ZEB1, SNAIL and TWIST) and EMT markers (N-cadherin and Vimentin), while E-Cadherin (an epithelial marker) was increased. In agreement with this, ADAM17 inhibitors such as ZLDI-8 were shown to prevent EMT in HCC cells by decreasing the release of Notch NICD, thereby improving the effects of anti-cancer drugs [137,138]. ADAM17 was also involved in transactivation of Notch signaling in liver cancer stem cells, contributing to an aggressive phenotype [116,139]. In addition, Notch signaling was implicated in the ADAM17-dependent activation of integrin β1 thereby promoting the migration and invasion of HCC cells [140]. In that context, interaction of ADAM17 with G-protein-coupled receptor 50 was suggested to drive the ADAM17-induced Notch signaling toward HCC progression [141]. While ADAM10 also activates Notch signaling, no report involving a Notch-ADAM10 signaling pathway to promote HCC progression has been reported yet, may be due to its ligand-dependency mechanism while ADAM17 activates Notch signaling by a ligand-independent mechanism [7]. However, ADAM10 promotes the proliferation, invasion and migration of HCC cells [34] and contributes to HCC progression through the shedding of CD147 (EMMPRIN or basigin), a transmembrane glycoprotein involved in metabolic adaptation of cancer cells, chemo-resistance and EMT [142].

The interplay between adamalysins and TGF-β-dependent signal might be a paradigm of the complex contribution of adamalysins activities in hepatocellular carcinoma. TGF-β plays a central role in chronic liver diseases, EMT and HCC progression [143,144]. We previously showed the association between ADAM12 expression and HCC aggressiveness and its regulation by TGF-β in hepatic stellate cells [36,145]. We next demonstrated that ADAM12 interacts with and stabilizes TGFBRII receptor, thereby promoting TGF-β signaling pathways through increase of receptor trafficking [9,146]. Using dedicated cellular models, we showed that ADAM12 promotes TGF-β-mediated EMT in a proteolytic independent manner [147]. ADAM12 also acts as a downstream player of EMT by regulating invadopodia and focal adhesion structures [148,149]. As an actor of TGF-β and Notch [8] signaling pathways, which are both involved in EMT, ADAM12 might enhance EMT by synergizing these pathways, although such a mechanism has not yet been addressed in the context of HCC progression. Beside ADAM12, we demonstrate that ADAMTS1 expression is increased in HCC and also contributes to amplify the TGF-β signal by stimulating the conversion of the latent-TGF-β into its active form in hepatic stellate cells [68,150]. Since HSCs are major cellular components of HCC stroma, where they modulate the proliferation and invasiveness of cancer cells [151], adamalysins expressed by HSCs are likely to play a pivotal role in these processes.

To conclude, deregulation of the expression of many adamalysins is associated with hepatocellular carcinoma. These have been implicated at all stages of HCC progression including inflammation, fibrosis, angiogenesis, proliferation, epithelial–mesenchymal transition and invasion offering broad clinical perspectives. However, their spatial and temporal dynamics of expression and their mechanisms of action are still insufficiently characterized. Future work should focus on better characterizing the contribution of the different hepatic cell types in the expression and activity of these new regulators of HCC progression.

## Figures and Tables

**Table 1:** Adamalysin expression in liver cancers from The Cancer Genome Atlas (TCGA) database. nd; not detectable; ns, not significant.

	T versus Adjacent NT Tissue (*n* = 48)		Kaplan–Meier Survival Analysis (*n* = 364)	
Name		*p*-Value	Worse Prognosis (Expression Low–High)	*p*-Value
ADAM2	nd	-	Low	2.00 × 10^−9^
ADAM7	nd	-	Low	1.40 × 10^−9^
ADAM8	ns	7.10 × 10^−1^	Low	1.20 × 10^−2^
ADAM9	increase	5.60 × 10^−13^	High	3.50 × 10^−4^
ADAM10	increase	7.20 × 10^−6^	ns	6.30 × 10^−2^
ADAM11	increase	1.50 × 10^−5^	Low	5.60 × 10^−3^
ADAM12	increase	2.20 × 10^−5^	High	4.00 × 10^−5^
ADAM15	increase	8.20 × 10^−19^	High	7.40 × 10^−3^
ADAM17	increase	2.10 × 10^−6^	High	3.70 × 10^−2^
ADAM18	nd	-	Low	8.30 × 10^−9^
ADAM19	ns	1.80 × 10^−1^	High	2.20 × 10^−2^
ADAM20	ns	2.00 × 10^−1^	Low	1.90 × 10^−3^
ADAM21	increase	1.50 × 10^−9^	ns	1.80 × 10^−1^
ADAM22	increase	4.80 × 10^−4^	ns	6.90 × 10^−2^
ADAM23	increase	1.30 × 10^−6^	ns	9.30 × 10^−2^
ADAM28	ns	8.90 × 10^−1^	ns	1.70 × 10^−1^
ADAM29	nd	-	Low	2.30 × 10^−9^
ADAM30	nd	-	Low	4.00 × 10^−8^
ADAM32	nd	-	Low	1.30 × 10^−2^
ADAM33	ns	5.90 × 10^−1^	Low	1.00 × 10^−3^
ADAMDEC1	increase	3.30 × 10^−3^	ns	2.70 × 10^−1^
ADAMTS1	decrease	5.90 × 10^−5^	ns	4.10 × 10^−2^
ADAMTS2	decrease	1.00 × 10^−4^	ns	4.80 × 10^−1^
ADAMTS3	ns	8.00 × 10^−1^	high	1.10 × 10^−2^
ADAMTS4	ns	7.80 × 10^−2^	ns	4.80 × 10^−2^
ADAMTS5	increase	2.40 × 10^−4^	High	1.00 × 10^−5^
ADAMTS6	increase	5.00 × 10^−03^	ns	1.70 × 10^−1^
ADAMTS7	increase	1.30 × 10^−15^	High	3.30 × 10^−2^
ADAMTS8	ns	2.30 × 10^−1^	Low	7.90 × 10^−4^
ADAMTS9	increase	2.60 × 10^−10^	High	3.20 × 10^−2^
ADAMST10	increase	6.10 × 10^−7^	Low	1.90 × 10^−3^
ADAMTS12	ns	2.60 × 10^−2^	ns	2.60 × 10^−1^
ADAMTS13	decrease	3.80 × 10^−21^	ns	6.70 × 10^−2^
ADAMTS14	increase	1.70 × 10^−6^	High	1.40 × 10^−2^
ADAMTS15	ns	7.10 × 10^−1^	ns	6.50 × 10^−2^
ADAMTS16	increase	8.40 × 10^−3^	ns	7.10 × 10^−2^
ADAMTS17	ns	2.20 × 10^−1^	Low	3.90 × 10^−2^
ADAMTS18	increase	9.30 × 10^−6^	Low	2.40 × 10^−2^
ADAMTS19	ns	-	Low	6.50 × 10^−9^
ADAMTS20	ns	-	Low	5.30 × 10^−6^
ADAMTSL1	ns	1.20 × 10^−1^	Low	1.90 × 10^−3^
ADAMTSL2	decrease	9.70 × 10^−6^	Low	3.30 × 10^−3^
ADAMTSL3	decrease	1.90 × 10^−2^	Low	1.10 × 10^−2^
ADAMTSL4	ns	1.20 × 10^−1^	Low	2.30 × 10^−2^
ADAMTSL5	increase	3.10 × 10^−5^	ns	6.80 × 10^−2^
ADAMTSL6 (THSD4)	ns	3.10 × 10^−1^	Low	1.40 × 10^−3^
